# Discrete choice experiments: An overview on constructing *D*-optimal and near-optimal choice sets

**DOI:** 10.1016/j.heliyon.2023.e18256

**Published:** 2023-07-18

**Authors:** Abdulrahman S. Alamri, Stelios Georgiou, Stella Stylianou

**Affiliations:** a School of Science, Royal Melbourne Institute of Technology University, Melbourne, VIC, 3000, Australia; b Department of Statistics, Faculty of Science, University of Jeddah, Jeddah, Saudi Arabia

**Keywords:** Multinomial logit model, Optimal designs, Efficient designs, Orthogonal main effect plans, Stated choice

## Abstract

Discrete choice experiments (DCEs) are frequently used to estimate and forecast the behavior of an individual's choice. DCEs are based on stated preference; therefore, underlying experimental designs are required for this type of study. According to psychologists, DCE designs consist of a small number of choice sets with a limited size in the number of alternatives within a choice set to increase the response efficiency in the questionnaire. Even though algorithmic constructions (known as efficient designs) become quite common for practitioners, optimal designs (sometimes so-called orthogonal designs) continue to be used in choice experiment studies, particularly in the case that prior information about the extent of the population preference is not available. Various approaches have been developed to construct DCE designs with fewer choice sets. However, the question in many practitioners' minds is which techniques perform better (i.e. given small designs with high efficiency) in a given circumstance. In this paper and to address these concerns, we conducted an overview of the constructions of discrete choice experiments in the literature for models with only main effects. The various ways of constructing optimal and near-optimal designs were compared in terms of their ability to minimize the number of choice sets in the survey. Our findings shed light on the optimal sample sizes needed for efficient experimentation which then can help the researchers to design more effective experiments in this area.

## 1 Introduction

A discrete choice experiment, sometimes so-called a choice experiment, is a type of stated preference method used in behavioral economics and market research to understand how individuals make choices. A Discrete Choice Experiment has commonly been conducted using the abbreviation DCE. It is often used when revealed preference data is not available or is not suitable for the research question at hand. DCE is based on a long-standing and well-tested theory of choice behavior, known as the random utility theory (RUT), which was proposed in [1]. DCE, unlike other “Stated Preference” SP approaches, enables the study of attribute interactions and involves hypothetical choices for respondents, which is a simpler and more realistic technique than ratings or rankings. It first arose as a means of determining customer demand for products and services that have not yet been traded on a market, e.g. having a new product under development and not yet available. DCE is helpful in making business decisions by assessing the importance of certain characteristics for their users in many different fields, such as marketing and manufacturing researchers [2], service providers [3], transport and logistics researchers [4], and policymakers [5], to name just a handful.

DCEs study mimic situations where people have to select from a variety of competing options based on their perceived utility to assess the influence of the attributes involved in choice options. Practically, each respondent is asked to repeatedly pick out the preferred scenario from a set of different competing options presented on each choice set. In your experiment, participants might be asked to answer all the questions that are included in the experiment, or they might be randomly assigned to answer only a particular subset of them, so-called blocking. Note that designs with a large number of choice sets can lead to poorer decision-making and a decrease in overall satisfaction with the decision. [6] mentions that if the complexity of the task increases, both the cognitive burden for respondents and random variability in responses are going to increase, whereas the efficiency of estimation will be reduced. As a result, it is important to carefully consider the amount and type of information presented in decision-making situations in order to optimize the quality of the decision. Therefore, the design of DCEs aims to strike a balance between minimizing the costs of data collection, reducing the cognitive burden on respondents, and maximizing the amount of valuable information gained, which can ultimately lead to more accurate modeling of complex decision-making processes [7].

**Figure 1:**
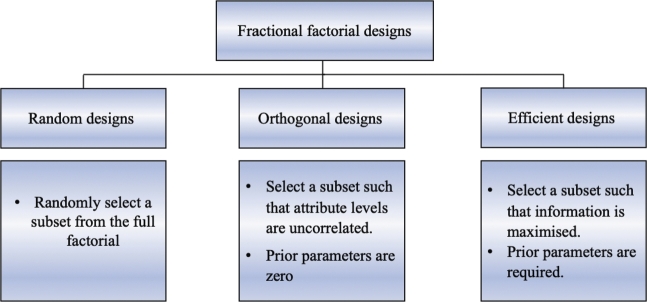
Experimental Design Methods.

Over the past years, there has been great interest from statisticians in researching the most effective methods for constructing optimal designs that somehow maximize the amount of information that can be obtained from the experiment while minimizing the number of runs required. One way to achieve this is by using fractional factorial designs, which are a type of design that allows practitioners to generate a subset of all possible combinations. There are different types of fractional factorial designs that can be used in constructing DCEs, Fig. 1 summarizes the differences between those types. These design strategies have been routinely adopted over the past years. Although recent studies have shown that efficient designs in which prior parameters are required become quite common for practitioners [8], still orthogonal and near-orthogonal designs which are only *D*-optimal in case the prior parameters are zero and all alternatives have the same probability of being chosen continue to be used in choice experiment studies, as these priors are usually not readily available [9]. For the purposes of this paper, however, D-optimal or near-optimal designs of choice sets maintain the orthogonality property, thus it maximizes the precision of the estimates of the model parameters, which is a major consideration from the researchers. For a more detailed description of the differences and advantages between constructing orthogonal designs and efficient designs, the previous review by Rose and Bliemer [10] provides in-depth information.

The literature on obtaining high-efficiency designs of DCEs with optimal reduced choice sets has received a lot of attention from researchers in this field since the late 20th and early 21st centuries. While previous research has covered this topic to some extent, we provide an extensive review in the area of DCE designs for models with only the main effect. Practitioners who specifically want to test for differences between respondents or samples need to use the same choice sets for all respondents, so they are drawn to designs with fewer choice sets to ensure that respondents complete them rationally. Although a lot of recent work on designs used in practice is based on algorithmic constructions, the focus of this paper will be on reviewing known theoretical approaches to designing optimal and near-optimal choice sets from various perspectives and insights. The various ways of constructing optimal and near-optimal designs were compared in terms of their ability to minimize the number of choice sets in the survey. Our findings shed light on the optimal sample sizes needed for efficient experimentation which then aid researchers in designing more effective experiments in this area. Additionally, we suggested directions for developing potentially new theoretical results on designs for DCEs.

The remainder of this article is laid out as follows. In Section 2, we introduce all the necessary definitions and notation. Then, in Section 2.2, we review the Street and Burgess *SB* approach including how to define a contrast matrix for the main effect only, and how to compare between different designs of choice sets using the *D*-optimality criterion. Then, in Section 3, we compile state-of-the-art direct construction methods from the literature for producing optimal and near-optimal DCE designs with main effects models. These are divided into two different cases, depending on whether the number of levels in all attributes is the same or mixed. Then, our main contribution lies in Section 4, where a comprehensive comparison of sample sizes was provided in terms of statistical efficiency and practicality (their ability to minimize the number of choice sets), given the relationship between the size of the choice set, the number of attributes, and their levels. Finally, in Section 5, we close with a discussion of construction techniques and areas for improvement and continued research.

## 2 Preliminary definitions and notation

**Figure 2:**
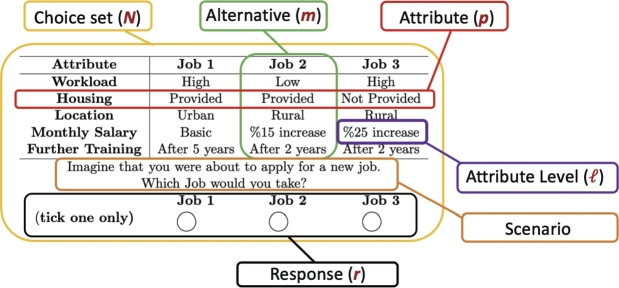
Notation and visualization of DCEs [11].

Throughout this section, we discuss several concepts that will be helpful in constructing optimal designs for the multinomial logit (MNL) model. Starting with some notation, we say that a DCE consists of a number of choice sets, denoted as *N*, and each choice set has two or more alternatives (m≥2), where each alternative is easily described by a set of *p* attributes (also known as factors), each attribute takes one of the *ℓ* levels, and these levels are represented numerically by *x*, x∈{0,1,...,ℓ−1}. In each choice set $T_{\alpha }$, model contains *m* different alternatives $T_{\alpha }=[T_{\alpha_{1}},T_{\alpha_{2}},\cdots ,T_{\alpha_{m}}]$, where $[T_{\alpha_{i}}\neq T_{\alpha_{j}}$ ∀ i≠j], and the $T_{\alpha_{i}}$ is the *i*th alternative in the *αth* choice set, α=1,2,...,N and i=1,2,...,m. Respondent *r* will choose the *ith* alternative in a choice set $T_{\alpha }$ if and only if $U_{r\alpha_{i}}\geq U_{r\alpha_{j}}$ ∀ j≠i, where *j* represents the other alternatives and $U_{r\alpha_{i}}=V_{r\alpha_{i}}+\epsilon_{r\alpha_{i}}$. $V_{r\alpha_{i}}$ denotes the systematic component of the utility that can be captured by the researcher and the $\epsilon_{r\alpha_{i}}$, so-called the unobserved components of the utilities, are independently identically Gumbel (known as type *I* extreme value) distributed (IID) with zero mode. Fig. 2 illustrates the notation and how a choice set, $T_{\alpha }$, would look like.

Throughout this paper, we consider designs under the multinomial logit (MNL) model for estimating the main effects since recent studies, such as [12], [8], showed an increase in the use of the MNL model for analyzing DCEs. It is worth noting that constructing choice designs under the MNL model, could be used to estimate many of those sophisticated models, e.g. panel mixed logit model, with a relatively high efficiency [13]. By contrast, many of these models can be recognized as generalizations of the MNL model, where the choice model is called MNL model if the $\epsilon_{r\alpha_{i}}$ is IID. It means that the respondents' choices in one choice set will not impact their choices in any other set of choices. Thus, the probability of choosing $T_{\alpha_{i}}$ in one choice set $T_{\alpha }$ is given by

$$Prob_{\alpha_{i}}=Prob(U_{\alpha_{i}}>U_{\alpha_{j}}\forall i\neq j)=\frac{\pi_{\alpha_{i}}}{\sum_{j=1}^{m}\pi_{\alpha_{j}}}=\frac{exp(\mu V_{\alpha_{i}})}{\sum_{j}exp(\mu V_{\alpha_{j}})}=\frac{exp(\mu \beta^{\prime }X_{\alpha_{i}})}{\sum_{j}exp(\mu \beta^{\prime }X_{\alpha_{j}})}$$

where $X_{\alpha }$ is the design matrix for respondent *α*, $\mu \beta^{\prime }$ is a matrix of estimable contrasts, with contrast coefficients in *μ* (where *μ* is a positive scale parameter usually assumed constant μ≡1) and $\alpha_{j}=1,2,...,m-1$ options presented to the respondent. Additional details about the assumptions of the MNL model compared to other alternative models can be found in [12], [14].

### 2.1 Assumptions

In this paper, we offer a condensed overview of the construction of optimal and near-optimal choice sets in the literature. As is known, the properties of DCE studies may vary from one design of choice experiment to another; thus, it would be useful to include studies that have similar aims in order to draw broad conclusions. Therefore, we assume in this paper the following; (i) a multinomial logit (MNL) model will be considered as a defining model used to analyze choice data. We have chosen the MNL model because it is the most commonly used model to analyze choice data [15], [16], allowing for an arbitrary choice set size and the only model used to evaluate the optimality of combinatorially generated designs; (ii) All interactions are negligible because our interest is only for the main effects; (iii) While there are multiple criteria for assessing optimal designs, for example, *D*-optimal, *A*-optimal, or *E*-optimal designs, the focus will be under the *D*-optimality criterion using the *SB* approach. *D*-optimality criterion is commonly used in practice and in literature due to its robustness against reparameterizations [17] and can be readily updated in cases where choice sets are added or removed from a DCE, which frequently occurs in algorithmic constructions [18]; (iv) All attributes are generic, i.e., partworth utilities are constant across all alternatives, and the choice sets are to be presented to respondents one at a time and they must choose one of the alternatives in each choice set. This approach in the literature is called Generic Forced-Choice Experiments [18]; (v) The attribute levels are not ordered in any way that would lead to the possibility of dominated alternatives, since orthogonal designs do not account for dominant alternatives.

Only studies that met or can be applied to these assumptions were included in this comparison. While some of the studies that will be discussed in this paper may not be well-known outside of the statistical community, they are considered by many experts to be superior to more commonly known methods. Thus, it is important to acknowledge the contributions of these researchers in this field and compared them to highlight the optimal reduction in the number of choice sets. Since a comparison of these designs will take place under the *SB* approach, we will review how the *SB* approach works to know how to select and group attributes into choice sets and how to evaluate between different choice experiments.

### 2.2 Evaluating DCEs using *SB* approach

The purpose here is to generate optimal or near-optimal choice sets, which give choices of *k*-tuple of attribute levels per task that optimize a stated criterion of interest for a certain total number of choice sets. This criterion is generally related to the information matrix of the design. Although Street and Burgess (SB) assume the MNL model for their designs, unlike other researchers, they use the previous approach, as it is described in [19], to derive the Fisher information matrix. Their matrix, so-called a *C*-matrix, is derived under the orthonormal coding with zero local priors and generic parameters across alternatives and is defined to $C=B\Lambda B^{\prime }$, where *B* is the contrast matrix for the effects to be estimated (i.e. main effects or main effects plus two-factor interactions), and Λ is the matrix of the second derivatives of the likelihood function. The Λ matrix is a symmetric square matrix of order *L* with rows and columns indexed by 0,1,...,L−1. To evaluate the entries in the Λ matrix, the occurrences of pairs of profiles are counted (see [19]) and the result is divided by $m^{2}N$. The diagonal entries of the matrix are chosen in such a way that the sum of each row and column is equal to zero. For more details on these, the interested reader is referred to [20].

#### 2.2.1 Contrasts

In the case of a quantitative variable, with $\ell_{q}$ levels, it is important to choose an appropriate coding scheme to represent the categories numerically. For a comprehensive understanding of the relationship between coding structure and design efficiency, the work in [21] provides valuable insights. Although “dummy coding” followed by “effect coding” consider the most common approach in coding the levels of a quantitative variable, one effective method for coding quantitative variables in DCE designs is to use a set of orthogonal polynomial contrasts (that is, the specific case examined by Street and Burgess as shown in equation (1)), which typically consists of $\ell_{q}-1$ contrasts. This approach is particularly suitable for capturing nonlinear relationships and can provide a more nuanced understanding of the impact of the factors being studied. While Street-Burgess (SB) examined the optimization of choice designs using orthonormal coding, it is necessary to demonstrate the construction of contrast matrices for the estimation of main effects exclusively. For simplification, we denote the contrast matrices for only main effects by $B_{M}$. The rows of the $B_{M}$ matrix are partitioned into a set of orthogonal polynomial contrasts represented by the polynomial contrast of order $\ell_{q}-1$ for each attribute, where $\ell_{q}-1$ is known as the degree of freedom of these effects. For example, for ℓ=3, contrasts-coded vectors will be represented by two rows, the entries of the first row will be −1, 0, and 1, corresponding to the polynomial contrast for the linear effects, and the entries of the second row will be 1, −2 and 1, corresponding to the polynomial contrast for the quadratic effects, and then normalized by dividing each row by the Euclidean norm ${\left\|\cdot \right\|}_{2}$, that is the square root of the sum of squares. Therefore, the corresponding information matrix for estimating the main effects is denoted by $C_{M}$ and is given by

(1) $$C_{M}=B_{M}\Lambda B_{M}^{\prime },$$

where $B_{M}\times B_{M}^{\prime }=I$. Note that $B_{M}^{\prime }$ is the transpose of $B_{M}$ and *I* is the identity matrix of size $p(\ell_{q}-1)$, q=1,...,p. Thus, the $B_{M}$ matrix is of order p(ℓ−1)×L when all attributes have the same number of levels or of order $p(\ell_{q}-1)\times L$ when attributes have a different number of levels. Bear in mind that the matrix $B_{M}$ is not unique for ℓ≥3, thus a change in contrasts does not change the outcome of the determinant $det(C_{M})$ as long as the matrix $B_{M}$ is normalized [22]. When we have higher number of levels we can use the method that is described in Chapter 4 of [23] for coding up to 10 attribute levels. The optimization of choice designs using linear effects coding can be found in [24] as an alternative for those who would like to investigate the differences between the two coding schemes. Note that deriving the information matrix of choice experiments under the linear effects coding or under the orthonormal coding are both equivalent for the purpose of finding optimal designs.

#### 2.2.2 Comparing designs

When comparing designs for DCE, there are several ways to evaluate their performance. One common approach is to compare the efficiency of the designs, which can be measured by various criteria such as the *D*-, *A*-, or *G*-criterion. Often, these criteria, are related to the information matrix of the design, previously mentioned as the $C_{M}$ matrix. In our case, we will only define the criteria based on the determinant, known as the *D*-efficiency, with respect to the estimation of the main effects. The notation $C_{opt,M}$ will be used to denote the information matrix for estimating the main effects of a *D*-optimal DCE.

A DCE design is said to be *D*-optimal if “it minimizes the generalized variance of the parameter estimates, that is, $det(C_{M}^{-1})$ is the smallest possible for the *D*-optimal designs” [20]. Indeed, a DCE design which minimizes $det(C_{M}^{-1})$ is the same as the one which maximizes $det(C_{M})$ [15], [25]. Thus, the *D*-error of a design is ${\left[det(C_{M}^{-1})\right]}^{1/\omega }$, and the *D*-efficiency of a design, denoted by $D_{eff}$, is given by;

$$D_{eff}={\left[\frac{det(C_{opt}^{-1})}{det(C_{M}^{-1})}\right]}^{\frac{1}{\omega }}={\left[\frac{det(C_{M})}{det(C_{opt})}\right]}^{\frac{1}{\omega }},$$

where *ω* is the number of parameters that are to be estimated in the model. For the main effects model we have that, $\omega =\sum_{q=1}^{p}(\ell_{q}-1)$ give a number to this equation. Remembering that $det(C_{M}^{-1})=1/det(C_{M})$.

The *D*-efficiency can be used as a guide so that any possible designs with different numbers of choice set *N* can be compared to a common reference scale [15]. Burgess and Street identified the *D*-optimal DCE from amongst the various designs under the assumption of equal choice probabilities. They provided an upper bound for $det(C_{M})$ for estimating the main effects for any choice set size with any number of attributes each having any number of levels. The determinant of the optimal design matrix $det(C_{opt,M})$ (the maximum value of the determinant of $C_{M}$) for a particular value of *m* is given in equation (2).

(2) $$det(C_{opt,M})=\prod_{q=1}^{p}{\left[\frac{2S_{q}\ell_{q}}{m^{2}(\ell_{q}-1)L}\right]}^{\ell_{q}-1}.$$

where

(3) $$S_{q}=\left\{ \begin{array}{cc} (m^{2}-(\ell_{q}x^{2}+2xy+y))/2, & \text{if}\;(2\leq \ell_{q}<m),\\ m(m-1)/2, & \text{if}\;(\ell_{q}\geq m), \end{array}\right.$$

and the positive integers *x* and *y* satisfy the equation $m=\ell_{q}x+y$ for $0\leq y<\ell_{q}$. By using equation (3), the value $S_{q}$ signifies the maximum number of differences in the levels of the attribute *q* in each choice set. It should be noted that *D*-optimal designs are not necessarily unique and, in particular cases, may not exist. In the next section, we provide some of the efficient techniques for constructing optimal and near-optimal DCE designs and models with only main effects.

## 3 Optimal and near-optimal choice sets

Many researchers sought to find techniques that can produce optimal and/or near-optimal designs of choice experiments that are easier for practitioners to apply and, at the same time, easier for subjects (respondents) to complete them. While it is not yet possible to provide direct constructions of globally *D*-optimal designs for every combination of attributes and runs, the methods we discuss here cover nearly all the practical cases for symmetric attributes (the same number of levels in all attributes) and asymmetric attributes (which have a different number of levels). Some of these methods are very specific and others are more broad-ranging. Under the assumptions included in Section 2.1, Table 1 provides all the construction information of optimal and near-optimal DCE methods for models with only main effects. It is worth noting that some of these papers, such as [27] and [16], identified the optimal designs under a different model called the linear paired comparison model. Others have suggested the use of A-optimality in linearized models for DCE designs, see for example [32]. However, under the indifference assumption of equal choice probabilities and for designs with only sets of paired choice, Großmann and Schwabe [15] observed that any optimal design under the MNL model is also optimal under the linear paired comparison model and vice versa.

**Table 1:**
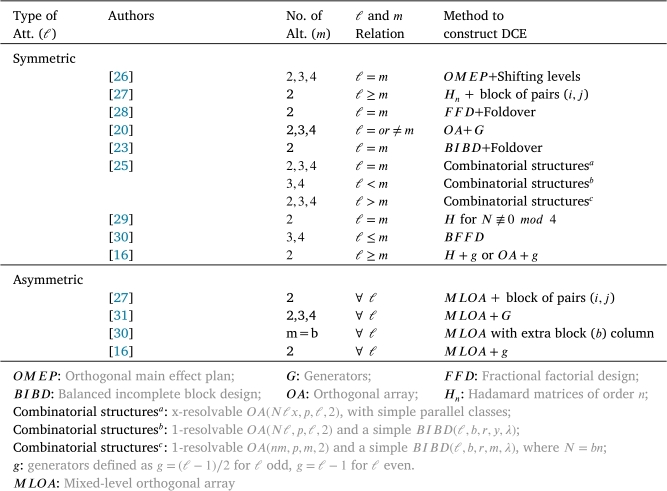
Construction information of optimal and near-optimal DCE methods for models with only main effects.

**Figure 3:**
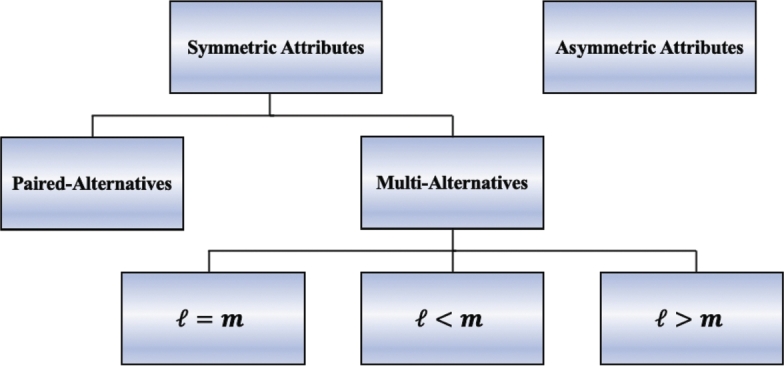
Structure of construction *DCEs*.

Usually, the main concern of practitioners is to find the most appropriate design with a manageable number of choice sets that can fit their experiments. However, the design construction involves several aspects and issues that may be related either to the appropriate size, e.g. the size and/or the number of choice sets that should be included, or to how the options are selected and grouped into choice sets, e.g. randomly or in order. In this section, we describe nearly all the methods used to construct these designs with a sufficiently small number of choice sets, some of which are specific to symmetric attributes, while others can handle asymmetric attributes. Fig. 3 shows the structure of construction DCEs for this section depending on the type of attribute levels and the size of choice sets in relation to the number of levels (i.e. ℓ=m, ℓ<m or ℓ>m).

### 3.1 Symmetric attributes

In DCEs, symmetric attributes play a crucial role in designing efficient and balanced choice sets. Explicit constructions of optimal or highly efficient designs with practical numbers of choice sets have been developed. Whilst it is possible to construct certain approaches utilizing designs that exceed four alternatives in size (m>4), it is important to note that doing so may result in a higher demand for information processing, which in turn could potentially impact the quality of responses provided by the respondent [33]. Also, Researchers, e.g. Großmann and Schwabe [15], stated that the use of choice sets of size smaller than five (m<5) is more effective in applications, since increasing the size of the choice sets further may lead to diminishing improvements. Thus, in this section, the focus will be on constructing the choice sets of size m=2,3, or 4. Note that all techniques that are included in our study of multi-alternatives can also work with more than four alternatives, i.e. m>4.

#### 3.1.1 Paired-alternatives

A Pair-Alternatives is a type of choice experiment in which participants must choose between pairs of alternatives depending on their perceived value. A simple method for generating optimal choice designs with choice sets of size m=2 is based on an orthogonal main effect plan (OMEP), in which all choice sets are as different as possible from each other with level balance and minimal overlap. OMEP design usually uses as an initial design (specifies one alternative in a pair) whereby the generation of the second alternative across all choice sets is based on the first alternative. This can happen when we use techniques such as “Shifting” [26], “Foldover” [26], [28] or “Generators” [16], [20]. Note that OMEP is based on regular FFDs with a resolution of III or higher (that is, the specific case examined in [28]). An excellent summary and introduction to OMEPs can be found in [34].

The foldover technique, often regarded as one of the initial methods employed in the generation of choice experiments, is commonly used to estimate the main effects of optimal designs for scenarios where the number of alternatives equals two. The technique of foldover, also referred to as “swapping levels”, yields a *D*-efficiency value of 100% for choice pairs consisting of *p* binary attributes when only testing the main effects. The optimal form of the $C_{M}$ matrix for the case when *N* is multiple of 4, i.e. (N≡0 mod 4), is

$$C_{M}=\left(\begin{array}{ccccc} \frac{1}{2^{p}} & 0 & 0 & \cdots & 0\\ 0 & \frac{1}{2^{p}} & 0 & \cdots & 0\\ \vdots & \vdots & \vdots & \ddots & \vdots \\ 0 & 0 & 0 & \cdots & \frac{1}{2^{p}}. \end{array}\right)$$

The maximum value of the determinant of $C_{M}$ was introduced and proved by Street and Burgess [28], and it is equal to $det(C_{opt,M})={\left(1/2^{p}\right)}^{p}$. Note that, for every attribute, the level in the second alternative is represented by a level that was not utilized in the first alternative. Thus, the *D*-optimal designs are in the case where all attribute levels are different in each pair. For more details see construction 1 in [28].

Simultaneously, Graßhoff et al. [27] have derived optimal designs for the symmetric case, but under a different model, called the linear paired comparison model. Their method, denoted by $G.1_{2004}$, involves the use of Hadamard matrices (that is, n×n square matrix with all elements equal to ±1 and satisfies $H_{n}H_{n}^{\prime }$ = $H_{n}^{\prime }H_{n}$ = $nI_{n}$). Note that it is particularly useful when all attributes have the same number of levels. The construction of $G.1_{2004}$ used *k* columns of a Hadamard matrix, denoted by $h_{1},...,h_{k}$, where the order *n* is the smallest integer which is a multiple of 4 and ≥*k*. For example, if the number of attributes is five p=5, then the chosen $H_{n,k}$ matrix will be the $H_{8,5}$ matrix, i.e., the constitution of any 5 columns of a Hadamard matrix of order 8. These columns are then combined with a (ℓ−1)ℓ/2×(ℓ−1) matrix $A_{\ell }$. Then the pairs are obtained from the matrix $H_{n,k}⨂A_{\ell }$. The total number of pairs in $G.1_{2004}$ is defined as N=n(ℓ−1)ℓ/2 and the generated design has a *D*-efficiency of 100%. For example, if we have five attributes (p=5) with three levels ($\ell_{q}=3$), then the needed run is eight r=8, and therefore N=24. We refer to [27] for construction details.

A DCE can also be generated from a binary BIBD with parameters v,b,r,k,λ, denoted by BIBD (v,b,r,k,λ), and its complement design, denoted by BIBD $\left(v^{\prime },b^{\prime },r^{\prime },k^{\prime },\lambda^{\prime }\right)$ [23]. However, A DCE optimal design will only be applicable when the BIBD designs are on this combination of parameters $v=s^{2}$, b=s(s+1), r=s+1, k=s, λ=1, so-called an affine resolvable BIBD. A BIBD with the parameters (9,12,4,3,1), for example, may be used to assign the 9 attributes to 12 2-alternative choice sets, where its complement (9,12,8,6,3) represents the second alternatives. Furthermore, DCEs can be generated with a fixed size using a BIBDs with parameters v,b,r,k,λ, where v=p= the number of attributes, b=N= the number of choice sets (blocks) of uniform size *k*, *r*= the number of choice sets in which each of the *p* attributes appears, k=m= the size of choice sets, and *λ*= the number of choice sets in which each pair of profiles appear together [26]. In this case, a BIBD with the parameters (9,12,4,3,1) may be used to assign the 9 attributes to 12 3-alternative choice sets. However, one practical limitation of BIBDs is the lack of availability for every possible combination of parameters v,b,r,k,λ. For more details regarding this approach, see [23] or [26].

A group of researchers consisting of Singh and Chai, Das and Dey, have been extremely active since 2015 and continue to be so in generating efficient DCE designs. Nevertheless, the design strategies explored by these researchers are less well-known outside of the mainstream statistical literature. Hence, the inclusion of the research undertaken by this group, while arguably superior to some of those discussed herein in terms of practicality (their ability to minimize the number of choice sets), would greatly benefit in obtaining designs that have the optimal reduction in the number of choice sets and maintain favorable efficiency.

Several constructions in which practitioners can construct optimal two-level paired choice designs for any number of choice sets *N* were suggested in [29]. Their method was based on Hadamard matrices, where extra columns and rows would be removed as needed to get the desirable optimal designs. Their constructions work more effectively in the unsaturated case, where N≠p and N>p. Furthermore, Dey et al. [16] have extended the $G.1_{2004}$ approach to overcome the large increase in the number of choice sets when *p* and/or *ℓ* increase. In their approach to the construction of DCE designs, they used Hadamard matrices and orthogonal arrays, denoted by $D.1_{2017}$ and $D.2_{2017}$, respectively. They constructed *D*-efficient paired choice designs with 30−50% fewer choice pairs. Their strategies are based on a collection of generators after finding a suitable starting design, while the number of generators is defined as g=(ℓ−1)/2 or g=ℓ−1 for odd or even, respectively. The total combinations, choosing 2 out of the *ℓ* levels, are $T_{c}=\ell (\ell -1)/2$. For more details on how to define the group combinations, see Lemma 2.1 in [16]. In particular, only $sg^{\prime }$ group combinations are used to obtain a *D*-efficient design, where $g^{\prime }=g-1$ or $g^{\prime }=g-2$ with each row of $\left[H_{n,k},-H_{n,k}\right]$ and s=ℓ or s=ℓ/2 for odd or even, respectively. The only group combinations that can be used to generate the choice pairs are $G_{i}(mod\;g)$, where $i=j-1,j,\cdots ,j+g^{\prime }-2$ and *j* correspond to the *j*th row. Hence, the total number of choice sets is defined as $N=nsg^{\prime }$ [16]. The $D.1_{2017}$ approach works effectively, with high *D*-efficiencies, for large attribute levels. i.e. ℓ=5,6,7,⋯, while with smaller levels it is at par.

#### 3.1.2 Multi-alternatives

In the real world, choice problems usually consist of more than two choices, which makes the use of multi-alternatives (m≥3) more realistic, e.g. buying a new product typically requires selecting one of the multiple alternatives. Another advantage of the multi-alternatives over the paired choice sets is that a choice experiment with larger sizes provides more details with fewer questions on respondents than can be derived through paired choice sets. However, the process becomes more sophisticated especially when ℓ≠m, where it may require one, or more than one, set(s) of generators to satisfy this condition (ℓ≠m). This technique, which requires a set of generators, is usually applied if there are no other effective approaches. Therefore, we group the methods to construct *D*-optimal choice sets into the following three categories based on the size of the choice set *m* in relation to the number of levels *ℓ*:

• **Case 1:** ℓ=m
• **Case 2:** ℓ>m
• **Case 3:** ℓ<m

Note that none of the cases 1–3 are *D*-optimal unless the condition,“for each attribute *q*, 1≤q≤p, the multi-set $Q_{q}$ contains each of the *ℓ* levels exactly Nm/ℓ time” is satisfied [25].

• **Case 1**

For the case ℓ=m, most of the known methods for constructing DCEs are applicable. In this article, however, we only include constructions that give the fewest possible choice sets.

Shifted approach, contrary to “Foldover”, uses the mathematical idea of modulo arithmetic, i.e., it adds a constant to all attribute levels of the initial *k* columns to generate one or more additional alternatives. The additional alternatives are always taken modulo *ℓ* since all attributes have the same levels (e.g. add 1 mod *ℓ*). Bunch et al. [26] stated that “the modulo arithmetic “shifts” the original columns in such a way that all attributes take on different levels from those in the original alternative”. The shifted approach is very effective when the number of attribute levels is equal to the size of the choice sets (i.e. ℓ=m). This can also be considered a limitation since when ℓ≠m the method requires finding the needed generators. The method that requires generators will be discussed later. For instance, if ℓ=m=3 and the original alternative is coded 02120, then one can shift the original alternative once to create the second alternative 10201 and one can be shifted twice to create the third alternative 21012 (i.e. add 1 and 2 modulo 3, respectively, to all attribute levels in the original alternatives). Shifting the attribute levels of the OMEP, denoted by $B_{1996}$, gives a *D*-efficiency of 100% for the designs of *p* symmetric attributes when only testing the main effects [26]. Subsequently, the $B_{1996}$ approach is simple to construct, contrary to the Difference Vectors approach from [35]. Under this case, ℓ=m, both approaches produce the same number of choice sets, while the Difference Vectors approach requires that “for each $v_{j}$ present, the bound of equation (3) is satisfied, and there is at least one $v_{j}$ with a non-zero $a_{v_{j}}$; that is, the choice set is non-empty” [31]. The Difference Vectors construction approach is worth being mentioned, as it is an efficient approach to constructing DCEs, and it will be further discussed later in the asymmetric attributes section.

In addition, four different approaches for constructing *D*-optimal symmetric choice sets were given in [25]. All their approaches highly reduce the number of choice sets, however, only two approaches will be mentioned in this paper, one under case 1 and one under case 2. These approaches produce designs with the smallest number of choice sets. In particular, their constructions involve the use of several combinatorial structures, such as BIBD and *OA*. An orthogonal array OA(R,p,ℓ,t) is defined to be an R×p array with elements from a set of *ℓ* levels such that any R×t subarray has each *t*-tuple appearing as a row an equal number of times, where *R* refers to the number of runs, *p* refers to the number of attributes (columns), *ℓ* refers to the number of levels, and *t* is the strength of the array.

The first approach of Demirkale et al. [25], denoted by $DC.1_{2013}$, is based on resolvable orthogonal arrays. The choice sets of $DC.1_{2013}$ consist of *N* simple parallel classes of $A_{i}$, where $A_{i}$ be an *x*-resolvable OA(Rm,p,ℓ,2) and each $A_{i}$ represents a choice set $T_{i}$ of size m=xℓ, i=1,⋯,R. If an OA(R,p,ℓ,t) consists of a set of *xℓ* rows, this design is said to be an x-parallel class, which means that each of the *ℓ* levels occurs in each column of the subarray *x* times, whereas if its rows can be partitioned into *x*-parallel classes, this design is said to be an x-resolvable class [25]. Note that a simple parallel class exists if no two rows are identical [25].

Recently, blocked fractional factorial designs, denoted by BFFD, were applied in [30] to construct DCEs with symmetric attributes. Unlike other blocking approaches, all respondents here face the same choice tasks. For a given $\ell^{p-k}$ FFD, it is divided into $\ell^{q}$ blocks to represent the number of choice sets in a DCE, with blocks of size $\ell^{p-k-q}$ to represent the number of alternatives in each choice set, where *p* is the number of attributes, *k* is the number of design generators for an FFD, *q* is the number of blocking variables (so-called block generators), ℓ=2 or 3 attribute levels. Additionally, only a resolution III FFD is needed, since we only want to estimate the main effects, and thus all aliased two-factor interactions are considered negligible. For instance, a one-quarter fraction of the full factorial design for five two-level attributes ($2^{5-2}$ FFD) can be constructed in two blocks (2^1^) each of size four ($2^{5-2-1}$). Hence, this design consists of two choice sets each with four alternatives and the design generators are: $X_{4}=X_{1}X_{2}$, $X_{5}=X_{1}X_{3}$, and one block generator is needed; $b_{1}=X_{2}X_{3}$. However, this approach has a limitation on the size of choice sets, as that must be a power of the number of attribute levels, e.g., using two-level BFFDs, that is, $2^{p-k-q}=2,4,8,\cdots$, using three-level BFFDs, that is, $3^{p-k-q}=3,9,\cdots$.

• **Case 2**

For the case ℓ>m, we will discuss the well-implemented approach in [25]. This approach is denoted by $DC.4_{2013}$. They used a 1-resolvable orthogonal array OA(nm,p,m,2) and a simple BIBD(ℓ,b,r,m,λ), to systematically decrease the numbers of the included choice sets. Note that a BIBD is called simple if no two blocks are identical. Their construction has an advantage over the construction of Theorem 2 of [36]. The $DC.4_{2013}$ approach can give designs with a larger range of values of *ℓ* for each *m*, instead of the construction in [36] that has some restrictions on the number of levels that can appear given the choice set size [25]. For example, if m=3, then *ℓ* can only be ℓ=3,4,7 in the construction of [36]. However, in the $DC.4_{2013}$ approach, each block of the *BIB* design will be written in *n* simple parallel classes of $A_{i}$, i.e. each block, labeled by $D_{q}$, 1≤q≤b, will have a set of choice sets (*n*) that gives a total of N=bn choice sets each of size *m* on *p* attributes each having *ℓ* levels. The next example will illustrate the above concepts.

• **Case 3**

In the literature, there are several approaches that give *D*-optimal or near-optimal designs for DCEs in the case we have ℓ<m. Therefore, in this case, an orthogonal array plus a collection of generator sets will be used to reduce the number of choice sets. In this approach, an orthogonal array, denoted by *OA*, is used as the starting design in [37] and [38], rather than the complete factorial design, denoted by *F*, which was proposed by [31]. Since our focus is only on estimating the main effects, the appropriate starting designs only need to be an orthogonal array of strength 2, denoted by $OA(R,\ell^{p})$ (i.e., the resolution of the design is III). Such orthogonal arrays can be found on Neil Sloane's website, http://neilsloane.com/oadir/. The website uses a file oa.N.k.s.t.name to indicate an orthogonal array (a particular class of OMEPs) with *N* runs, *k* attributes (or factors), *s* levels (for each of the attributes), and strength *t* (equivalently resolution t+1).

We need to introduce some relevant notation, as these were used in *Theorem 3* in [31] and in *Theorem 3.1* in [38]. A collection of generator sets is added as $G_{\alpha }=\{g_{\alpha_{1}}=0,g_{\alpha_{2}},\cdots ,g_{\alpha_{m}}\}$, where α=1,⋯,τ and $g_{\alpha_{i}}\neq g_{\alpha_{j}},\forall \;i\neq j$. Note that sets of generators that have difference vectors satisfy the upper bound for the sum of the differences ($S_{q}$), given in equation (3), does not guarantee an optimal design [20]. It is assumed that the *i*th generator in the *α*th set of generators is the *p*-tuple, $g_{\alpha_{i}}=(g_{\alpha_{i_{1}}},g_{\alpha_{i_{2}}},\cdots ,g_{\alpha_{i_{k}}}),\forall \;i=1,2,\cdots ,m$. It is also assumed that the multiset of differences for attribute *q* $\left\{\pm (g_{\alpha_{i_{1q}}}-g_{\alpha_{i_{2q}}})\;|\;1\leq i_{1},i_{2}\leq m,i_{1}\neq i_{2}\right\}$ contains each non-zero difference modulo $\ell_{q}$ equally often. Then the choice sets given by the rows of $OA+g_{\alpha_{1}},OA+g_{\alpha_{2}},\cdots ,OA+g_{\alpha_{m}}$, for α=1,⋯,τ, are *D*-optimal for the estimation of main effects only if it is provided that there are as few zero differences as possible in each choice set [31]. Using equation (3), denoted by $S_{q}$, we get the largest possible number of pairs of profiles that can have different levels for attribute *q* in a choice set. For a set of generators, for given values of *m* and $\ell_{q}$, there are exactly m(m−1)/2 pairs of positions for each attribute in each choice set. For the *q*th position in the *p*-tuple, the corresponding entry in the difference vector equals to 0 if $(g_{\alpha_{i_{1q}}}-g_{\alpha_{i_{2q}}})=0$, and equals to 1 otherwise [38]. Thus, repeated values are allowed.

Despite the strength of the OA+G approach, the lack of a solid mathematical background may constitute an obstacle for practitioners in determining the best generators [15]. Street and Burgess [20] admit that when this method is used “it is something of an art to obtaining the smallest design”. Thus, a general result that gives the optimal set of generators still remains a challenging problem.

### 3.2 Asymmetric attributes

Throughout the previous sections, we discussed the design of optimal and near-optimal choice sets of DCEs with symmetric attributes. In this section, we will illustrate some appropriate approaches that can be applied to reduce the size of DCEs with asymmetric attributes for the estimation of the main effects only. However, mixing the levels of different attributes will increase the number of choice sets required to achieve attribute level balance. For example, if we have three attributes with two, three, and five levels, respectively, then the lowest possible number of choice sets that will balance these levels is 30 choice sets. As a result, it is best not to mix too many different numbers of attribute levels, unless they all have even or odd levels of attributes [10]. For example, assume that we now have three attributes but with two, four, and six levels, respectively; then we will only need 12 choice sets to balance these levels (since this is the lowest possible divisible number by two, four, and six).

Various strategies are available to construct mixed-level orthogonal arrays. The interested reader is referred to the comprehensive review of orthogonal arrays and their constructions using mathematical algorithms and their implementation in C codes [39] and SAS macros [40], or using theoretical constructions [41], [42], [43]. A drawback of these strategies is that there is no indication that a design is the best (optimal) design. Therefore, to date, a strategy of using mixed-level orthogonal arrays seems to be the most effective method for constructing DCEs with asymmetric attributes. However, a particular orthogonal array may not be available each time, so practitioners have no efficient alternative approach other than using *OA* with more levels than needed and then collapsing these levels to get the levels they need. Keeping in mind that before the choice sets are constructed, the collapse of levels needs to be implemented in the starting design. For more details, see Chapter 8 in [20].

This approach has no restrictions in terms of the number of attributes and the size of the choice set, as long as the needed orthogonal array exists. This approach is similar to the previous approach, mentioned in case 3, with the difference that the starting design will be a mixed-level orthogonal array. Then the choice sets are generated in a similar way either by adding one or more sets of generators. This approach is general and produces optimal designs to estimate the main effects only as long as those generators have the maximum number of level changes for each attribute [37].

Researchers are advised to be cautious in the case of constructing a DCE design, and at least one of its attributes has no prime number of levels, i.e. has a composite number, that may cause some difficulties in constructing an efficient design. For example, constructing paired choice sets with asymmetric attributes while a particular attribute has four levels, hence, at maximum, only four out of the six possible pairs can be generated for this particular attribute. Thus, using several sets of generators across the same starting designs to generate the final design would be the only efficient solution available to date [37], [44]. For such a design, one generator can consist of 2 (*mod* 4) and another of either 1 or 3 (*mod* 4).

A general approach, was recently presented in [44], to determine the possible number of generators that can be used to construct paired choice sets, rather than a trial-and-error approach. The following Theorem 3.1 presents a simple result that systematically provides the *h* generators;

Theorem 3.1 *[44]* *The number of generators for the optimal paired choice design with p attributes is* $h=lcm(h_{1},...,h_{q})$ *where* $h_{q}=\ell_{q}-1$ *for* $\ell_{q}$ *even and* $h_{q}=(\ell_{q}-1)/2$ *for* $\ell_{q}$ *odd,* q=1,⋯,p *. The generators are then given by* $G_{\alpha }=(g_{\alpha_{1}},g_{\alpha_{2}},...,g_{\alpha_{q}})$ *, where* $g_{\alpha_{q}}$ *takes each of the values from the set* $1,...,h_{i}$ *with frequency* $h/h_{i}$ *,* α=1,⋯,h *,* i=1,⋯,k *.*

Note that $lcm(h_{1},\cdots ,h_{q})$ denotes the least common multiple of $h_{1},\cdots ,h_{q}$, however, the sets of generators given by Theorem 3.1 may not give the optimal reduced choice sets [44], N=h×R. Thus, this needs further investigation in the future.

## 4 Comparison of approaches sample sizes

In order to move the study forwards and open up new areas of inquiry, it is essential to conduct a comparison of approaches that have been constructed in this field. Given the above cases, each time we have included at least one technique to generate optimal or near-optimal choice sets for the designs of *p* symmetric attributes. However, some techniques have the ability to be applied to more than one case with very high efficiency, and most of the time equal to 100% using an *SP* approach, but usually with a different number of sets of choices in a given circumstance. In this section, a comparison of the sample sizes of the mentioned techniques in relation to the size of the choice set, the number of attributes, and their levels are attempted to estimate the main effects only. This is applicable under the assumption that all the respondents should face the same choice tasks, in order to highlight the optimal reduction in the number of choice sets. Also, we will delve into the aspects where certain approaches exhibit limitations, shedding light on their constraints and potential challenges.

Table 2 presents the smallest possible number of choice sets for *D*-optimal paired choice designs, DCEs, which were derived using construction techniques based on: reduction of the shifting approach from OMEP as starting designs, given by [26]: reduction of the Hadamard structure given by [27], denoted as $G.1_{2004}$: reduction of the Foldover approach from FFD given by [28], denoted by $SB_{2004}$: reduction of an x-resolvable orthogonal array, given by [25], denoted as $DC.1_{2013}$: reduction of a resolvable orthogonal array and simple BIBD, given by [25], denoted as $DC.4_{2013}$: reduction of an orthogonal array of strength two plus generator/s, a collection of generator sets is given by [31], and denoted by OA+G.

**Table 2:**
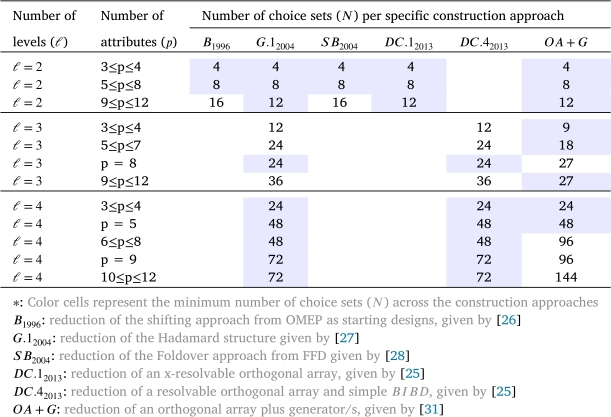
A comparison of approaches for *D*-optimal DCEs for m=2.

The number of choices in the available optimal paired choice designs increases rapidly as *p* and/or *ℓ* increase even moderately. Because such large designs are not really appealing to a researcher, designers such as [20] and [16] suggested some techniques to construct small designs with reasonably high *D*-efficiencies for the estimation of the main effects. Attributes with up to four levels ℓ=4 have been displayed in Table 2, whereas the same powerful techniques in Table 3 were given by [16], denoted by $D.1_{2017}$ and $D.2_{2017}$. Their techniques present about 30−50% reduction in the number of choice pairs at the cost of 1−15% loss in *D*-efficiency, but still with higher *D*-efficiencies against the existing orthogonal designs, such as the designs in [20]. In Table 3, the number of generated choice sets from techniques in [16] will be compared with the smallest possible number of choice sets. These techniques give an optimal DCE with *ℓ* varying from 4 to 7 as mentioned in Table 2. The results of this table were first shown in [16], but here they are presented differently to make it easier for the reader to see the differences among all the designs included in this review.

**Table 3:**
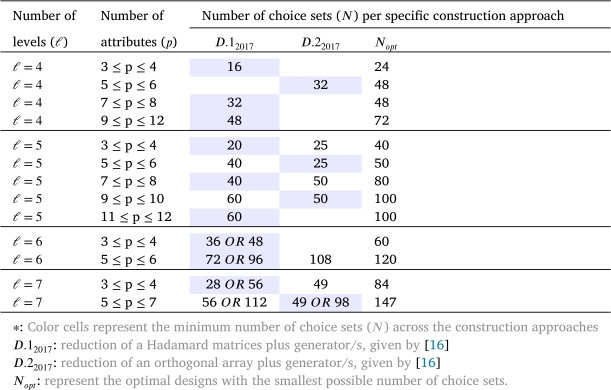
A comparison of approaches for near-optimal DCEs for m=2.

Table 4, Table 5 present the smallest possible number of choice sets for *D*-optimal choice designs of triple and quadruples size for DCEs, respectively, which derived using the construction techniques based on: reduction of the shifting approach from OMEP as starting designs, given by [26]: reduction of block fractional factorial designs given by [30], denoted as BFFD: reduction of an x-resolvable orthogonal array, given by [25], denoted as $DC.1_{2013}$: reduction of a resolvable orthogonal array and simple BIBD, given by [25], denoted as $DC.4_{2013}$: reduction of an orthogonal array of strength two plus generator/s, a collection of generator sets are given by [31], and denoted as OA+G.

**Table 4:**
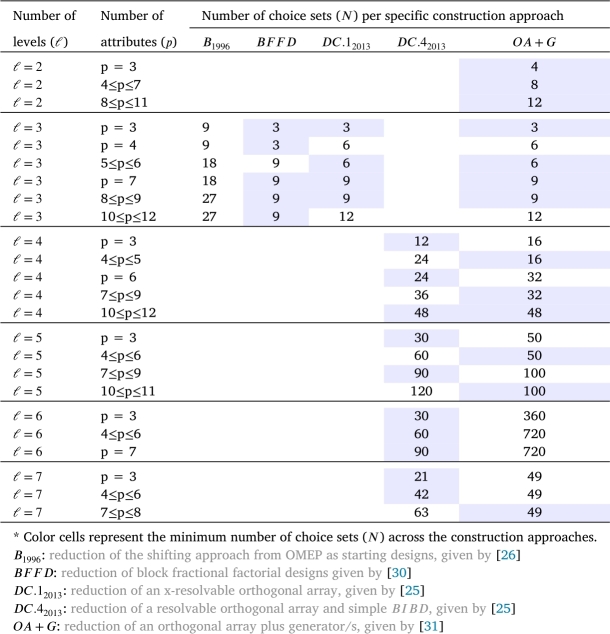
A comparison of approaches for *D*-optimal DCEs for m=3.

**Table 5:**
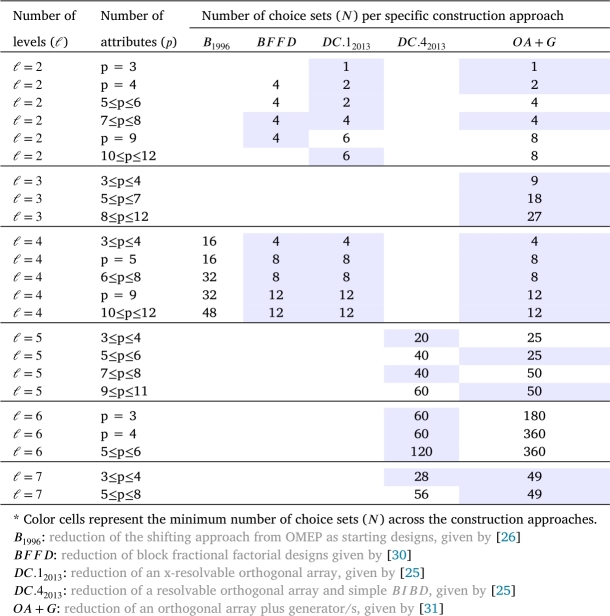
A comparison of approaches for *D*-optimal DCEs for m=4.

In light of the information presented in the previous Table 2, Table 3, Table 4, Table 5, it is evident that certain approaches may be better suited for certain cases than others. Some of these cases are listed here. Approaches, such as $G.1_{2004}$, $SB_{2004}$, $D.1_{2017}$ and $D.2_{2017}$, are only applicable to construct an optimal DCE design in the case when the size of choice sets is two m=2. On the other hand, approaches, such as $B_{1996}$, $DC.1_{2013}$, $DC.2_{2013}$, and OA+G, are suitable to construct an optimal DCE design in all three cases of the size of choice sets. In addition, with regard to the size of the choice set and the number of attribute levels, the approaches $B_{1996}$ and $SB_{2004}$ are limited to construct an optimal DCE design in the case when the size of choice sets is equal to the number of attribute levels ℓ=m. Conversely, the approach labeled as $DC.4_{2013}$ is only appropriate for generating a DCE design when ℓ>m. Finally, in relation to the previous findings, the BFFD approach is able to be used when the size of choice sets is three or four (m=3 or 4).

Upon conducting a critical review, it becomes apparent that there exist certain inherent constraints and challenges associated with these approaches. The present text enumerates certain constraints and challenges. A drawback of the foldover technique, as considered in the study conducted in [28]), is that attribute levels are only paired with its foldover companion code, e.g., if ℓ=4 and an alternative is coded 0123, it will be paired with 3210, or if ℓ=3 and an alternative is coded 21100, it will be paired with 01122. Thus, some alternatives cannot occur, and hence, optimality cannot be achieved either. The “Foldover” technique also has the drawback of becoming increasingly challenging to manage as the number of attribute levels increases. Thus, the “Foldover” technique is only effective in the case when the number of attribute levels and the size of the choice sets is both two (i.e. m=ℓ=2). Conversely, the approach labeled as $B_{1996}$ performs more effectively once the size of the choice sets is equal to the number of attribute levels (i.e. m=ℓ), otherwise identifying the right generators (that is, the specific case examined by [20]) is necessary to construct an optimal DCE design with fewer choice sets. Lastly, in relation to challenges, the number of choice sets increases rapidly as the number of levels or attributes increases, see Table 2.

Turning to another aspect of comparison, there are notable differences between the approaches in terms of their ability to minimize the number of choice sets in each given circumstance. For example, when m=2, the results show that the $G.1_{2004}$ approach and $DC.4_{2013}$ approach tend to perform better when the number of levels is even, otherwise, the OA+G approach is more effective in generating a DCE design with fewer choice sets. The main advantage of the $G.1_{2004}$ approach is that it can be easily applied to construct attributes with higher levels using Hadamard matrices since Hadamard matrices are known to exist for all *r* multiple of 4 up to n≤664. However, the $G.1_{2004}$ approach has the issue of quickly increasing the number of choice sets when levels or attributes increase. The reduction in the number of choice sets is typically achieved in one of two ways. Some researchers developed in a side where the choice sets are constructed using the generators, as in [16] and [20], while other researchers tend to increase the size of choice sets, as in [25] and [30].

Table 3, Table 4, Table 5 shows a number of ways in which optimal and near-optimal designs for DCEs with fewer choice sets can be obtained. The results in Table 3 show that the $D.1_{2017}$ approach tends to perform better than the optimal reduction in the number of choice sets, especially with a larger number of attribute levels. i.e. ℓ=4,5,6,7,⋯. According to the findings presented in Table 4, Table 5, the OA+G approach consistently exhibits superior or comparable performance to the BFFD approach in cases where *ℓ*= or <*m*. Conversely, the $DC.4_{2013}$ approach consistently generates designs with fewer choice sets when ℓ>m.

The tables were obtained by collecting and combining the information about the existing designs from various scholarly sources, with the aim of trying to put the parameters and the properties of the existing designs together. This will provide a direct access to what designs are available in the literature. By identifying the parameters of the needed designs (p,ℓ,m), the practitioner can efficiently locate the desired design and its corresponding explicit construction through the provided references. The main result demonstrated by these tables is archiving designs with the optimal reduction in the number of choice sets and maintaining favorable efficiency. These provide options that the practitioners could access directly.

## 5 Discussion and ongoing work

In this study, we reviewed several efficient techniques for generating DCE manually in order to show the differences between the DCE approaches from different perspectives. Studies, such as [12], [8], identified that the number of DCE applications using *D*-efficient designs has continued to increase, being applied in more and more countries to comprehend and evaluate individual preferences. Therefore, this paper provided the most appropriate approaches that result in *D*-optimality or high *D*-efficiency design of DCEs with optimal reduced choice sets. We found that most of the known techniques can be applied to only symmetric attributes, whereas only *OA* can be applied to both symmetric and asymmetric attributes. For symmetric attributes, we grouped the methods for constructing *D*-optimal choice sets into three categories based on the size of the choice set *m* in relation to the number of levels *ℓ*. For each category, we display different direct construction methods, providing practitioners with an enormous toolbox of designs and design construction methods available to be used in their work. There is no other publication that we are aware of that has such a comprehensive list of construction methods, as well as the specific method to utilize and any necessary beginning vectors for each situation. We anticipate these methods to be the most useful for conditions in which ℓ>m or ℓ<m as these are the most complicated settings for finding the *D*-optimal design of DCEs with the smallest possible number of choice sets.

As in most previous reviews, the majority of DCEs used a fractional design rather than a full factorial design, and within this class there exist many different types of design. For example, as mentioned in Health Economics, 74%, 89%, 55%, and 89% of the DCE applications used a fractional design in the periods 1990-2000 [45], 2001-2008 [14], 2009-2012 [12] and 2013-2017 [8], respectively. However, the number of attributes, the number of attribute levels, and the size of choice sets, i.e. the design parameters, always play a crucial role in the choice of the appropriate techniques for the construction of DCEs. They are required to be specified in the early stages of the experiments, since the information matrix is based on the parameters of the MNL model [30], [46]. Predominantly, a potential problem with practitioners, after identifying the parameters (the utility functions), is to find the most appropriate designs, of manageable size, that can fit the parameters of interest with high-efficiency [7], [20]. In this article, we reviewed how optimal or near-optimal designs of DCEs can be constructed using various efficient techniques from the literature. The assumption for all methods was that all alternatives per choice set are equally attractive. In the sequence, these techniques were compared in specific instances of parameters.

First, we found that not all construction techniques can be applied to all cases; i.e. some of the above techniques can only be applied to symmetric attributes, whereas others can be applied to both symmetric and asymmetric attributes. To the best of our knowledge, no available techniques yet can be applied to both symmetric and asymmetric attributes other than the *OA* approach with one of the previously mentioned techniques, such as *OA* plus generators. However, the orthogonal array still has some limitations; some of the *OA*s may have the same number of runs for different parameters, and for other cases, the needed orthogonal arrays may not exist [16], [20]. Thus, constructing optimal and near-optimal choice sets using orthogonal arrays for the only main effects model, is subject to the availability of the required orthogonal array *OA*s and the existence of the optimal set of generators. Some suitable orthogonal arrays have been highlighted in [38].

Also, we noticed that not all construction techniques can satisfy all the desirable DCE properties and, at the same time, have high efficiency. Thus, constructing designs satisfying as many as possible of the desirable DCE properties are completely desirable. Street an Burgess [20] acknowledged that “minimizing the number of choice sets could be the first priority and that such a design, with somewhat lower efficiency, is perfectly acceptable”. For example, in a paired choice design, an optimal choice sets design usually offers a large number of choice sets, more than a respondent can complete. However, the $D.1_{2017}$ techniques offer designs consisting of 30−50% fewer choice sets with near-optimal performance, i.e., designs have high *D*-efficiencies. Thus, the $D.1_{2017}$ techniques work sufficiently when the number of attribute levels involved is large, such as ℓ=5,6,7,..., while with smaller levels they are at par; see Table 3. However, the $D.1_{2017}$ technique has the limitation that the given number of generators that are needed to generate the final design may not always be the smallest possible, especially when the design has small attribute levels, such as ℓ=2,3,4 [16].

Additionally, some designs that we included in this article can only be used for a specific size of the choice set, e.g. paired, triple, or quadruple choice sets; otherwise, they are not suitable to be used or they produced a larger number of choice sets compared to other techniques. For example, the technique of [27] is denoted by $G.1_{2004}$ and the techniques of [16] are denoted by $D.1_{2017}$ and $D.2_{2017}$, are only suitable for paired choice sets, while the Shifting approach, denoted as $B_{1996}$, could be applied to more than one specific size of the choice set. Note that the $B_{1996}$ approach often produces a larger number of choice sets in comparison to other techniques. For example, when p=4,ℓ=2.m=2, we would need 8 choice sets in order to have 100% efficiency, whereas the optimal choice sets at the same parameters using other approaches, e.g. the $G.1_{2004}$ approach or the OA+G technique, we only need 4 choice sets in order to have 100% efficiency. Therefore, we suggest that if the size of the choice set is two, m=2, and looking for an optimal choice set design that offers the smallest number of choice sets, then the $G.1_{2004}$ technique prefers to be used when *ℓ* is an even number, and the OA+G technique prefers to be used when *ℓ* is an odd number. However, when *ℓ* is higher than 4, we recommend using $D.1_{2017}$ or $D.2_{2017}$ as they offer designs with fewer choice sets. A future proposal here may be whether odd sizes m=3,5,7,⋯ may perform better than even sizes m=2,4,6,⋯ on people's responses? This could be investigated either mathematically, experimentally by psychologists in human behavior, or by surveys asking people's opinions. Collaborative work with psychologists and qualitative researchers could be proven useful in addressing this problem.

On the other hand, we identified that some approaches are good with a small number of attribute levels, but when either the attributes or the attribute levels increase, the produced designs that need to be presented to respondents are becoming large and unattractive to practitioners. To illustrate that, for parameters, ℓ=4,P=4,m=3, the OA+G technique produces an optimal DCE with 16 choice sets, whereas, for parameters ℓ=6,P=4,m=3, a DCE design with 720 choice sets are generated with the same technique. Similarly, a DCE constructed using the [25] technique, denoted as $DC.4_{2013}$, produces DCEs with choice sets 24 and 60, respectively. In general, the OA+G technique often produces fewer distinct choice sets as long as the number of attribute levels is less than or equal to the size of the choice sets (ℓ≤m), otherwise, when (ℓ>m), the $DC.4_{2013}$ technique produces fewer choice sets in most cases.

In terms of reducing the size of choice sets in the questionnaire, we could also use blocking techniques that were applied in a variety of ways by many researchers, see for example [20], [29], [44]. These authors allocated the alternatives of preference (choice sets) into more than one block either randomly or using a spare attribute. Keep in mind that orthogonality will be lost in the data if the blocks are not balanced [10]. Note that, other than this, blocking is not relevant due to the assumption that all people (respondents) are similar. However, DCE designs were constructed in [44], by using blocked optimal paired choice sets, instead of the traditionally choice designs as was done in [20], [47]. The respondent heterogeneity was treated as a nuisance factor and included it in the model in [44]. It was noted by [48], that the respondents of the *D*-optimal design, which is constructed by [44], can be heterogeneous as they may be influenced in choosing from the alternative in a pair, as a result of how the options are presented or arranged with the pair. Bearing in mind that some of the choice sets of the design given to a respondent may have replicated alternatives, i.e. contains the same alternatives *s* and *t* twice in different orders, i.e. (s,t) and (t,s). Hence, choosing a different way makes sense when the respondent's choices are influenced by the order of the alternatives, otherwise, it sounds odd [48]. One future proposal comes from [44] and in that paper the authors suggested performing a comparison between their own results with the results of [49].

In the comparison of the sample sizes, we observed that some of the presented approaches produce optimal DCEs with fewer distinct options depending on the size of choice sets in relation to the number of levels (i.e. m=ℓ, m>ℓ or m<ℓ). In Table 4, Table 5, for instance, it is clear that when *m* is equal to *ℓ* or *m* is less than *ℓ*, techniques such BFFD, $DC.1_{2013}$, and OA+G produce DCEs with fewer distinct choice sets. On the other hand, when *m* is greater than *ℓ*, the $DC.4_{2013}$ technique produces fewer choice sets than any other technique in most cases. While a large range of practical settings is covered by these constructions, several miscellaneous issues exist, especially when there is a high number of attributes and/or levels. Note that a small number of attributes and/or levels do not appear to be “realistic” [15].

Under the main effects model, the case of *D*-optimal choice sets for the designs of *p* binary attributes has almost been completely covered in the literature either using paired choice sets [15], [27], or at larger sizes of choice sets [25], [35]. For the design with 3≤k≤12 symmetric attributes, i.e. all attributes have the same levels, the most efficient approaches so far were suggested by [25] at arbitrary sizes. However, some techniques of [25] and [27] have recently been modified by [16] to construct paired choice designs with 30−50% fewer choice pairs than existing designs and, at the same time, have reasonably high D-efficiencies for the estimation of the main effects.

It is worth mentioning that in this article the number of choice sets, *N*, is mostly zero modulo 4, i.e. N≡0mod4, whereas some practitioners somehow may need to use choice sets that are of a size other than zero modulo 4, i.e. N≢0mod4. Some researchers might argue that there is no need for constructing DCEs having other than N≡0mod4 choice sets, as both optimality and orthogonality are achieved in this case. Others argue that there is a need to construct designs with N≢0mod4 for practical applications. In paper [17] the authors pointed out that “the whole field of optimal design theory is predicated on the belief that experimental designs should tailor to the needs of the practitioner and not the other way around”, which means practitioners should not be forced to fall into the N≡0mod4 case. Another potential criticism from researchers may be that it is easy to generate the required choice sets, with N≢0mod4, as there is software that is able to do so using sophisticated search algorithms, such as SAS macros and Ngene. However, in the same paper they also claimed that “these algorithms do not guarantee global optimality and may take substantial computation time to converge upon an answer”. To the best of our knowledge, the only article available in the literature to design choice sets that is not a multiple of four, N≢0mod4, is [29]. In that paper, the authors only talked about optimal two-level choice designs of paired choice sets. Nevertheless, the techniques in [29] have a lack of research since they give no specific guidelines on how to determine which columns to either select or delete. As such, this still remains an open and interesting area of research.

In conclusion, the purpose of this paper was to provide the most efficient techniques for constructing optimal and near-optimal choice sets of main effects only for academics and practitioners to use in their work. These techniques were compared using the *D*-optimality criterion under the assumption that all alternatives per choice set are equally attractive. Moreover, the presented design techniques are only applicable to be applied when the choice experiment is unlabeled. A multinomial logit (MNL) model was assumed because it could be used to estimate all sophisticated models with relatively high efficiency. Future work in the discrete choice experiments could be to either modify some different initial designs or construct a new initial design to generate improved designs, as new work related to these suggestions by the authors of this paper will be finalized and published soon. Also, a future proposal may be to find alternative experimental designs to construct DCEs with optimal or near-optimal choice sets, and that would be a useful practical contribution.

## Additional information

No additional information is available for this paper.

## CRediT authorship contribution statement

All authors listed have significantly contributed to the development and the writing of this article.

## Declaration of Competing Interest

The authors declare that they have no known competing financial interests or personal relationships that could have appeared to influence the work reported in this paper.

## Data Availability

No data was used for the research described in the article.
